# Comprehensive analysis of ferroptosis-related genes for clinical and biological significance in hepatocellular carcinoma

**DOI:** 10.1007/s12672-023-00677-4

**Published:** 2023-05-17

**Authors:** Qixian Wu, Zhenlin Tan, Yu Xiong, Chengxin Gu, Jingdon Zhou, Hui Yang, Jiyuan Zhou

**Affiliations:** 1 Department of Gastroenterology, the Second Affiliated Hospital of Guangzhou Medical University, Guangzhou, China; 2 Guangzhou Medical University, Guangzhou, China; 3 Intervention and Cell Therapy Center, Peking University Shenzhen Hospital, Shenzhen, China

**Keywords:** Hepatocellular carcinoma, Ferroptosis, Prognosis, CAPG, SLC7A11

## Abstract

**Objective:**

This study aims to build a prognostic model of hepatocellular carcinoma (HCC) with ferroptosis-associated genes and explore their molecular function.

**Methods:**

Gene expression data and clinical information were obtained from the Gene Expression Omnibus (GEO) and The Cancer Genome Atlas (TCGA) databases and the International Cancer Genome Consortium (ICGC). A ferroptosis-associated gene set was obtained from the FerrDb database to identify differentially expressed genes. Then, we performed pathway enrichment analysis and immune infiltration analysis. A combined model based on ferroptosis-associated genes for predicting the overall survival of HCC was built by univariate and multivariate Cox regression analyses. Quantitative real-time polymerase chain reaction, Western blotting, colony formation, CCK-8, and EdU incorporation assays were performed to clarify the function of CAPG in the regulation of cell proliferation in human HCC. Ferroptosis was evaluated by glutathione (GSH), malondialdehyde (MDA), and total iron detection.

**Results:**

Forty-nine ferroptosis-related genes were significantly correlated with HCC, 19 of which had prognostic significance. CAPG, SLC7A11 and SQSTM1 were used to construct a novel risk model. The areas under the curves (AUCs) were 0.746 and 0.720 (1 year) in the training and validation groups, respectively. The survival analysis indicated that patients with high risk scores exhibited worse survival in the training and validation groups. The risk score was also identified as an independent prognostic factor of overall survival (OS), which established and validated the predictive abilities of the nomogram. The risk score was also significantly correlated with the expression of immune checkpoint genes. In vitro data showed that CAPG knockdown dramatically suppressed HCC cell proliferation, and the underlying molecular mechanisms might be that the silencing of CAPG reduced the expression of SLC7A11 and promoted ferroptosis.

**Conclusion:**

The established risk model can be used to predict the prognosis of HCC. At the mechanistic level, CAPG may drive HCC progression by regulating SLC7A11, and ferroptosis activation in HCC patients with high CAPG expression may serve as a potential therapeutic strategy.

**Supplementary Information:**

The online version contains supplementary material available at 10.1007/s12672-023-00677-4.

## Introduction

Hepatocellular carcinoma (HCC) is one of the most common malignant tumors worldwide, leading to substantial morbidity and mortality [1]. In China, HCC is the fourth largest malignant tumor and the second largest cause of tumor-related death, posing a significant threat to the life and health of the Chinese [2, 3]. Despite the continuous improvement in traditional treatment methods such as surgical resection, radiotherapy, chemotherapy, and targeted therapy, the overall 5-year survival rate is only 18.1% [4]. Therefore, more studies on identifying useful biomarkers, improving the clinical treatment effect, and developing novel prognostic models are still urgently needed for the management of HCC patients.

Ferroptosis is a regulated form of iron-dependent cell death through the lethal accumulation of lipid-based reactive oxygen species (ROS) when the glutathione (GSH)-dependent lipid peroxide repair system is damaged [5]. Epidemiological and animal studies have shown that the Fe-rich microenvironment, which usually characterizes malignant tumors, supports rapid proliferation and promotes carcinogenesis [6, 7]. A high iron environment causes increased oxidative stress in cancer cells, leading to increased toxicity [8]. In addition, accumulating evidence for a mechanism by which ferroptosis suppresses cancer cells suggests that cancer cells are more sensitive to the induction of ferroptosis [9, 10]. Therefore, ferroptosis can serve as a specific and novel therapeutic strategy for cancer research. Recent studies have also identified that immune cells such as CD8^+^ T cells enhance ferroptosis-specific lipid peroxidation and contribute to the effect of immunotherapy [11]. The prognosis of lung adenocarcinoma and thyroid cancer is predicted by combining ferroptosis-related characteristics with the tumor immune microenvironment [12, 13]. Therefore, understanding the relationship between ferroptosis and the immune microenvironment may provide a more comprehensive perspective on cancer therapy.

Our study established and validated a ferroptosis-related prognostic model with high predictive power for HCC patients. We further performed pathway enrichment analysis of the key genes to study the underlying mechanisms. Additionally, we analysed the clinical value of the HCC risk score model in the immune microenvironment. Finally, we explored the biological function of ferroptosis-related key genes in developing HCC.

## Materials and methods

### Data sets and patients

The RNA-seq transcriptome profiles (fragments per kilobase per million, FPKM) of 374 HCC samples and 50 normal tissue samples were obtained from the TCGA-LIHC database (https://portal.gdc.cancer.gov/). Age, sex, tumor grade, TNM stage, follow-up time, and survival status were collected (Supplementary data 1). In addition, we used three datasets from GEO to extract a gene expression matrix for differential gene expression analysis (Supplementary data 2). (Total number of samples: 1077) GEO is a public functional genomics data repository that supports data submission according to the Minimum information about a microarray experiment (MIAME) standard, which accepts data based on arrays and sequences. The tools can help users query and download experimental and selected gene expression profiles. The information for the GEO dataset used is shown in Supplementary Table S1. The RNA-seq transcriptome profiles (FPKM) and clinical information of the external cohort for validation were obtained from LIRI-JP (260 samples) in ICGC (Supplementary data 3). The study conformed to the principles of the Helsinki Declaration.

### Identification and functional analysis of differentially expressed genes in ferroptosis

First, we selected three datasets, GSE25097, GSE45267, and GSE36376, in the GEO database. The datasets provided a gene expression matrix of normal and HCC samples, and then we analysed the differentially expressed genes (DEGs) (|log_2-_fold change (log_2_FC)|> 1, p.adj < 0.05, limma package) and displayed them in the form of volcano plots. A total of 213 ferroptosis-related genes (including drivers, suppressors, and markers) were identified based on the FerrDb database. The ferroptosis-associated genes obtained from the FerrDb database were experimentally verified. The DEGs obtained from the GEO datasets were intersected with the ferroptosis gene set to obtain differentially expressed ferroptosis genes. The results were displayed in the visual form of a Wayne diagram. The expression correlation of 49 genes is shown in the heatmap (Spearman's test). Based on the Metascape online analysis website, the 49 ferroptosis genes were analysed by Gene Ontology (GO) and Kyoto Encyclopedia of Genes and Genomes (KEGG) enrichment analysis (P values, hypergeometric test). To further capture the relationships between the terms, a subset of enriched terms was selected and rendered as a network plot, where terms with a similarity  > 0.3 are connected by edges. We selected the terms with the best p values from each of the 20 clusters. The network was visualized using Cytoscape (An Open Source Platform for Complex Network Analysis and Visualization), where each node represents an enriched term and is colored first by its cluster ID and then by its p value. For 49 genes, PPI enrichment analysis was carried out with the following databases: STRING, BioGrid, OmniPath, and InWeb_IM. Only physical interactions in STRING (physical score  > 0.132) and BioGrid were used (details). The resultant network contains the subset of proteins that form physical interactions with at least one other member. If the network contained between 3 and 500 proteins, the Molecular Complex Detection (MCODE) algorithm was applied to identify densely connected network components.

### Establishment and validation of the ferroptosis-associated prognosis model

The normalized messenger RNA (mRNA) expression data and patient information were obtained from TCGA-LIHC. Kaplan‒Meier (KM) analysis (overall survival, grouped according to the median gene expression level, the log-rank test) was performed on the 49 genes obtained, and further univariate Cox regression analysis was performed for the statistically significant part (*P* < 0.05). A total of 373 patients in the TCGA-LIHC cohort were randomly divided into the training group (n = 186) and the validation cohort (n = 187). Multivariate Cox regression analysis was carried out on the training group, and a risk score model based on ferroptosis-associated genes was built. The model results were analysed by KM analysis and verified by the validation group. In addition, we validated the signature in the external cohort LIRI-JP. Then, the risk model built in the training group was combined with common clinical indicators, such as sex, age, tumor stage, and tumor grade, to perform univariate and multivariate Cox regression analyses. We also used a time-dependent receiver operating characteristic (ROC) curve to test the efficiency of this model in predicting 1-, 3-, and 5-year survival.

We put the above results (risk score model) into the OS nomogram to visualize the prognosis. To test the prediction accuracy of the model at 1, 3, and 5 years, calibration analysis was carried out.

### Study on the relationship between the construction of the HCC risk score model and immune-related genes

In addition, since the previous GO/KEGG pathway analysis of 49 ferroptosis-related dysregulation genes has shown that they are involved in the immune regulatory system, the correlation analysis of three key genes, CAPG, SLC7A11, and SQSTM1, based on the TCGA-LIHC dataset showed that the expression of CAPG was highly correlated with each component of the cellular immune microenvironment (Spearman's test, ssGSEA). Accordingly, the immune score, stromal score, and ESTIMATE score were used to evaluate its prognostic significance. In addition, to explore the potential mechanism of ferroptosis-related key genes in HCC, we further analysed the relationship between the risk score model and the immune tumor microenvironment. First, considering the important role of the immunosuppressive microenvironment in a tumor, we obtained the immune checkpoint genes PD-1, CTLA-4, and TIM3 with negative regulatory function from the Tracking Tumor Immunophenotype (TIP) database. We also compared the differences in these negative regulatory genes between the high- and low-risk groups in the validation group and the training group based on the gene expression profile of the TCGA-LIHC dataset. Finally, we verified the findings in the external cohort LIRI-JP.

### Cell culture, transfection, and biological behavior assessment

The human HCC cell lines L02, Hep3B, and SMMC-7721 were purchased from the Chinese Academy of Sciences (Shanghai, China); HepG2 and SK-hep1 were obtained from American Type Culture Collection (Manassas, VA, USA); MHCC-97H, MHCC-LM3, and Huh7 cell lines were kindly provided by Southern Medical University. These cell lines were cultured in Dulbecco's modified Eagle's medium (DMEM) (Gibco BRL, USA) with 10% fetal bovine serum (FBS) (Biological Industries, Israel) and cultured in a humidified incubator with 5% CO_2_. The medium samples were stored at −80 °C. Cells were collected from the wells daily by trypsinization (Boster Biological Technology Co., Ltd., Wuhan, Hubei, China), and the cell numbers were determined with a hemocytometer. Small-interfering RNA (siRNA) against CAPG (si-1: sense 5'–3': GCA UUUCACAAGACCUCCATT; antisense 5'–3': UGGAGGUCUUGUGAA AUGCTT; si-2: sense 5–3': GGUGUGGUGGAAAGUCCAATT; antisense 5'–3': UUGGACUUU CCACCACACCTT) and the negative control (siCon) (sense 5'–3': UUCUCCGAA CGUGUCACGUTT; antisense 5'–3': ACGUGACACGUUCGGAGAATT) were constructed by GenePharma (Suzhou, China). RT‒PCR and Western blotting were used to test the knockout efficiency. Cell viability and proliferation were measured by CCK-8 (#34304, Selleck Chemicals, Shanghai, China), colony formation, and EdU (#C10310-1, RiboBio, Guangzhou, China) incorporation assays according to the manufacturer's instructions.

### RNA isolation and qRT‒PCR analysis

Total RNA was extracted using TRIzol reagent (Thermo Fisher, Inc., USA). Reverse transcription was performed with the PrimeScript RT Reagent Kit (TaKaRa Biotechnology, Dalian, China). Four replicates and three independent experiments were performed for each gene. GAPDH mRNA expression levels were used as internal normalized controls. Comparative quantification was performed using the 2-ΔΔCt method. The primer sequences are shown in Supplementary Table S2.

### Total protein isolation and western blotting

Proteins extracted from cell lysates were separated by electrophoresis on a 10% sodium dodecyl sulfate–polyacrylamide gel and transferred to nitrocellulose membranes by Bradford electroblotting according to the manufacturer's instructions (Sigma‒Aldrich). The antibodies were as follows: CAPG (#GTX114301-S, 1:200 dilution, Abcam), GAPDH (#60004–1-AP, 1:1000 dilution, Proteintech), SLC7A11 (#12691S, 1:1000 dilution, CST), and GPX4 (#52455S, 1:1000 dilution, CST).

### Iron assay

According to the manufacturer's instructions, the relative iron concentration in cell lysates was measured by an Iron Assay Kit (#ab83366, Abcam, Cambridge, MA, USA).

### Glutathione assays

The relative glutathione (GSH) concentration in cell lysates was assessed using a total Glutathione Assay Kit (#S0052, Beyotime Biotechnology, Shanghai, China).

### Lipid peroxidation assay

The relative malondialdehyde (MDA) concentration in cell lysates was assessed using a Lipid Peroxidation Assay Kit (#S0131S, Beyotime Biotechnology, Shanghai, China) following the manufacturer's instructions.

### Statistical analysis

All statistical analyses were conducted by using R software (version 4.2.1). Concurrently, Metascape, a gene annotation and analysis resource, and GraphPad Prism (version 8.0.1) also participated in the analysis. The exact *P* value or range was listed in the images to make judgments about statistical analysis results. The range of *P* values indicated that the difference was statistically significant: *P < 0.05; **P < 0.01; ***P < 0.001.

## Results

### Differentially expressed ferroptosis genes in HCC

**Fig. 1:**
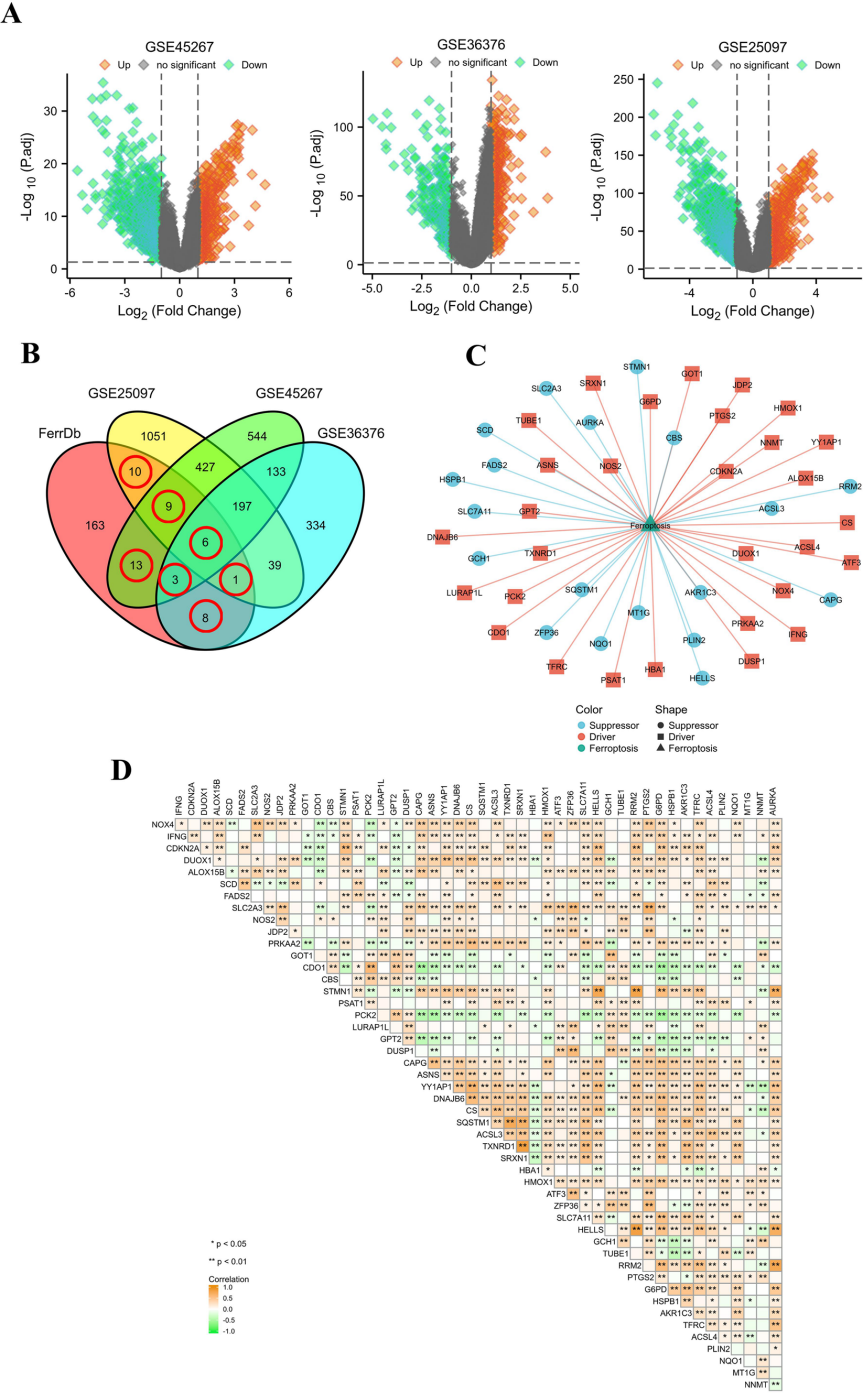
Overview of the differentially expressed ferroptosis-associated genes in HCC. **A** Volcano plots showing the differentially expressed genes in normal and tumor samples in the GSE45267, GSE36376, and GSE25097 datasets. **B** The Venn diagram shows the common differentially expressed genes among the four datasets. **C** The roles of 49 genes in ferroptosis. **D** Correlation of the expression of 49 genes is shown in the heatmap

We obtained 1749 abnormal genes from GEO: GSE25097, of which 610 genes were upregulated and 1139 were downregulated. A total of 721 abnormal genes were obtained from GEO: GSE36376, of which 450 genes were upregulated and 271 were downregulated. Finally, 1537 abnormal genes were obtained from GEO: GSE45267, of which 702 were upregulated and 835 were downregulated (Fig. 1A). The three datasets were intersected with 213 ferroptosis-related genes collected from the FerrDb database, and 49 DEGs (LOC284561 was deleted) related to ferroptosis were obtained (Fig. 1B). The detailed results are presented in Supplementary data 4. The roles of 49 genes in ferroptosis are shown in the network diagram. Thirty of them were drivers, and 19 were suppressors according to the FerrDb database (Fig. 1C)**.** The heatmap shows the expression correlation of 49 genes. Among them, RRM2 & STMN1, RRM2 & HELLS and SRXN1 & TXNRD1 were highly correlated. (Correlation coefficient r > 0.8) (Fig. 1D).

### Function analysis and network enrichment analysis

**Fig. 2:**
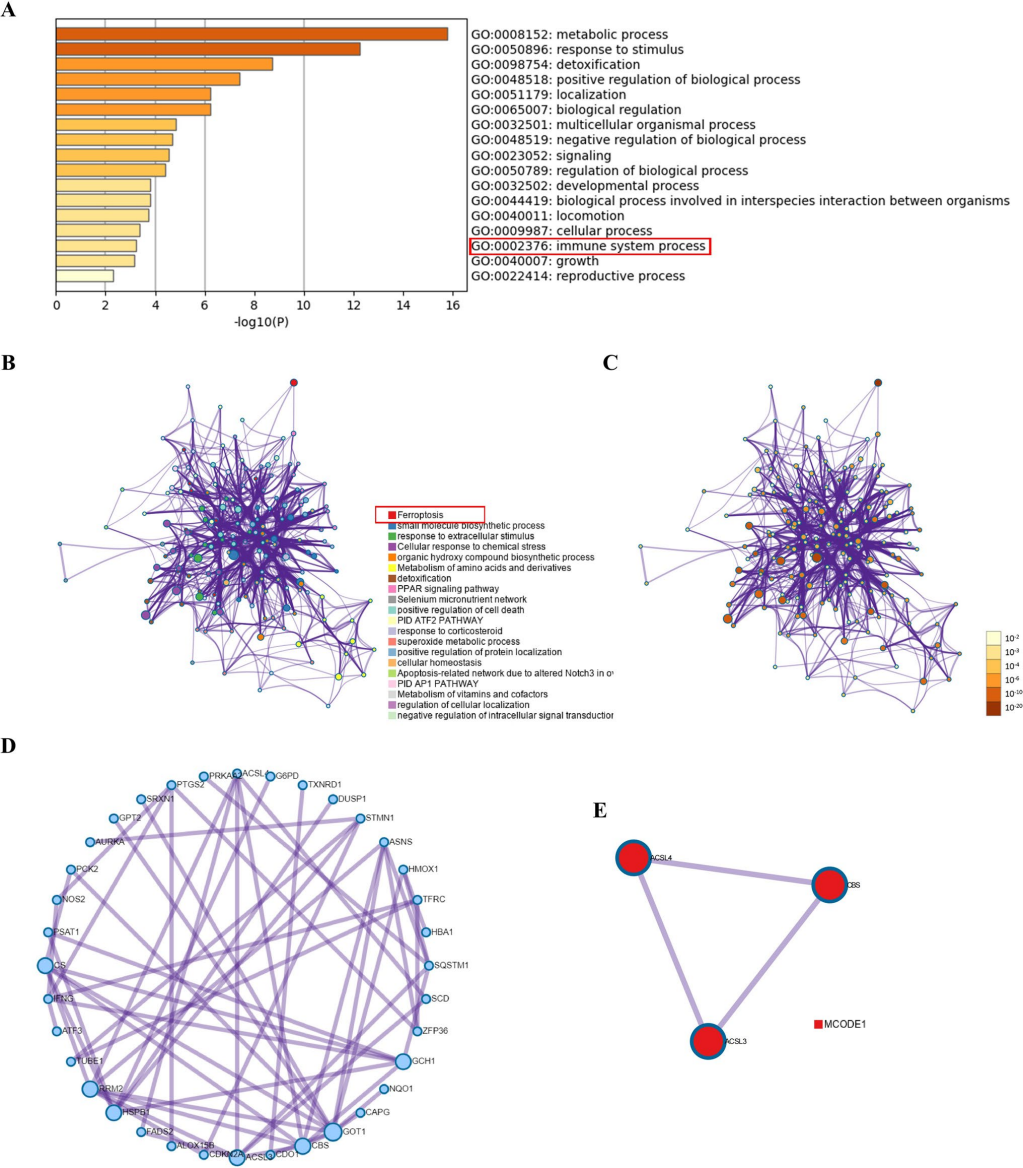
Functional analysis of the common differentially expressed genes **A** Bar graph of enriched terms, colored by p‒values. **B** Network of enriched terms, colored by cluster ID, where nodes that share the same cluster ID are typically close to each other. **C** Colored by p‒value, where terms containing more genes tend to have a more significant p‒value. **D-E** PPI network and MCODE components showing the hub genes in the ferroptosis gene set

The results showed that these differentially expressed ferroptosis genes were mainly enriched in response to oxidative stress, extracellular stimulation, immune system process, and molecular anabolism. KEGG pathway analysis showed that these differentially expressed ferroptosis genes were mainly concentrated in the PPAR signaling pathway, fluid shear stress, and atherosclerosis (Fig. 2A–C). These pathways might be important mechanisms that affect the prognosis of HCC patients. Based on the PPI network of Metascape-Online, we identified the important modules of these ferroptosis genes and showed the key genes of ferroptosis, including SQSTM1, CBS, GOT1, ASNS, RRM2, ACSL3, ACSl4, and CS (Fig. 2D). Pathway and process enrichment analysis was applied to each MCODE component independently, and the three best-scoring terms by p value were retained as the functional description of the corresponding components, as shown in the tables underneath the corresponding network plots (Fig. 2E).

### Construction and validation of the ferroptosis-related prognostic model

**Fig. 3:**
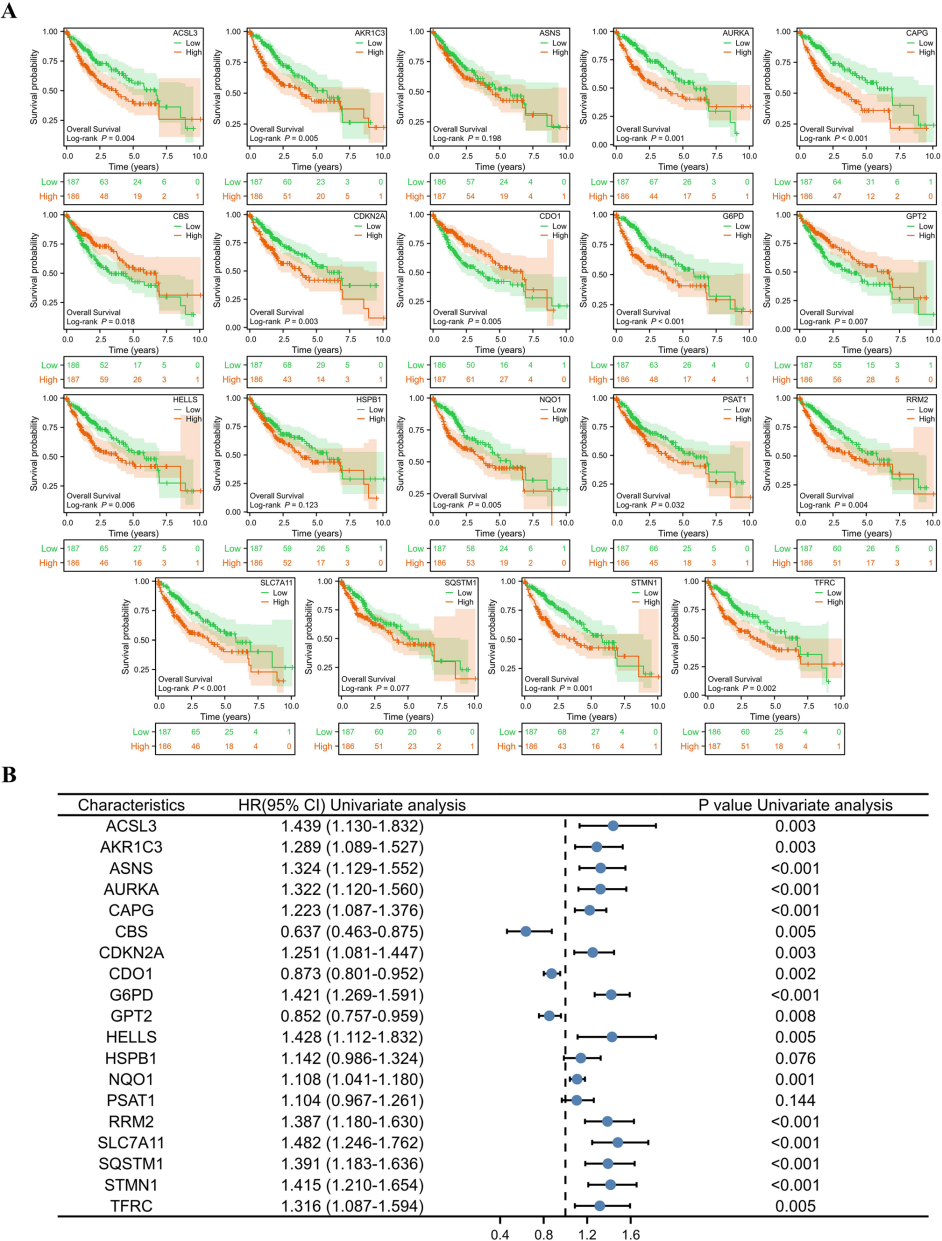
Kaplan‒Meier plots and forest plots of the prognostic ferroptosis genes. **A** Kaplan‒Meier plots show the 19 ferroptosis-associated genes with prognostic value. **B** The forest plot shows the results of the univariate Cox regression analyses of the 19 ferroptosis genes with prognostic value. *P < 0.05; **P < 0.01; ***P < 0.001

**Fig. 4:**
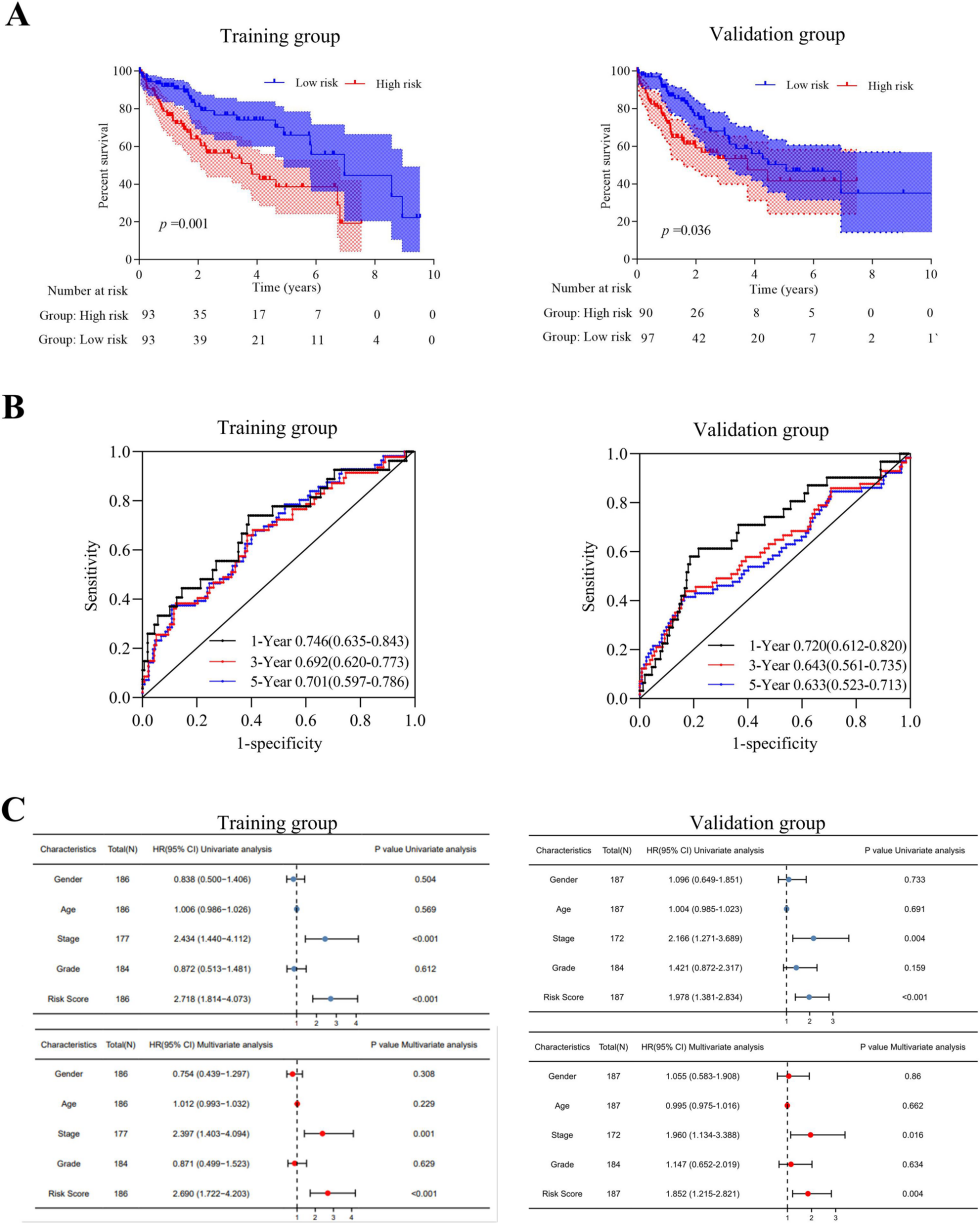
Establishment and validation of the ferroptosis-associated risk score model **A** The predictive model construction was based on multivariate Cox regression analysis. **B** The calculations of the model are according to the multivariate Cox regression analyses. **C** Univariate and multivariate Cox regression of clinical factors and risk score

Nineteen genes involved in ferroptosis were identified as prognostic genes, and 18 were statistically significant in univariate Cox regression analysis (Fig. 3A, B). Finally, CAPG, SLC7A11 and SQSTM1 were identified as independent prognostic signatures in a multivariate Cox regression (Table 1**)**. Therefore, the risk score was calculated as follows: 0.22 * CAPG + 0.44 * SLC7A11 + 0.24 * SQSTM1-2.44. Then, the high-risk group (n = 93) and the low-risk group (n = 93) were grouped by the median risk score. The median survival time of patients with high risk scores was significantly lower than that of patients with low risk scores in the training and validation groups (*P* = 0.001 and *P* = 0.036, respectively) (Fig. 4A). The predictive performance of the risk score for OS was evaluated by time-dependent ROC curves, and the AUC reached 0.746 at 1 year, 0.692 at 3 years, and 0.701 at 5 years in the training group; the AUC reached 0.72 at 1 year, 0.643 at 3 years, and 0.633 at 5 years in the validation group (Fig. 4B). The forest plots showed that stage and risk score were statistically significant (*P* < 0.001) (Fig. 4C). Validation in LIRI-JP further confirmed our conclusion (Supplementary Fig. S1). These results indicated that the prognostic model was well established.

**Table 1:** Multivariate Cox regression analysis of signatures in the TCGA-LIHC cohort

Variable	Coef	Exp (coef)	Se (coef)	Z	*P‒*value
CAPG	0.21730	1.24271	0.08583	2.532	0.01135*
SLC7A11	0.43820	1.54992	0.14819	2.957	0.00311**
SQSTM1	0.24209	1.27391	0.12409	1.951	0.04907*

### Clinical relevance investigation and nomogram construction

**Fig. 5:**
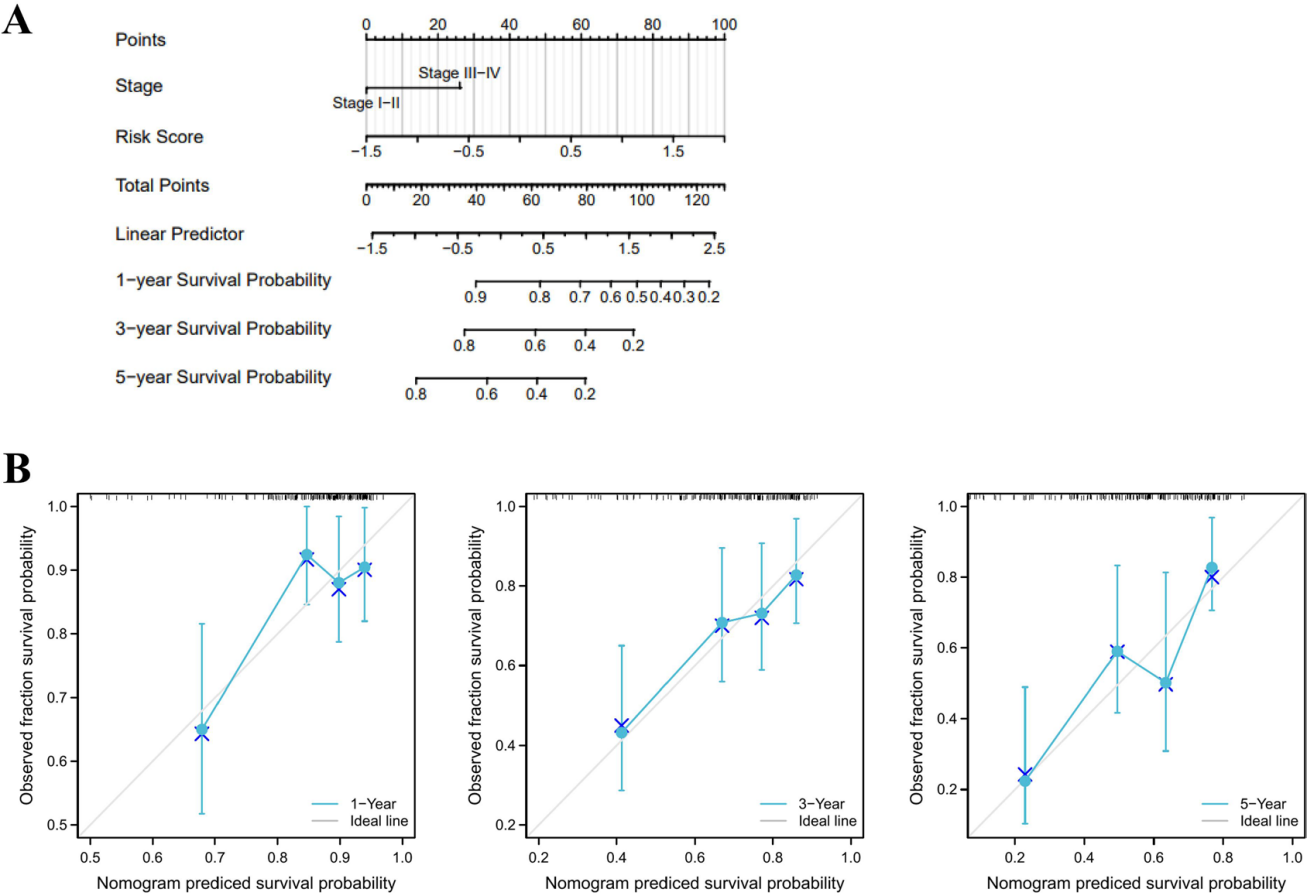
A clinical correlation between the risk score model and the prognostic nomogram for OS of HCC was established based on the risk score model. **A** The prognostic nomogram was established based on the risk score and clinical stage for prediction of 1-, 3-, and 5-year OS of HCC. **B** Calibration curve for the predicted probability of 1-, 3-, and 5-year OS

We used the results of multivariate analysis to establish a nomogram prognostic map associated with ferroptosis. We used a nomogram to predict 1-, 3- and 5-year OS (C-index = 0.672) (Fig. 5A). The nomogram can evaluate multiple variables to predict patient outcomes based on patient characteristics, including tumor stage and risk score. In addition, the calibration curve showed the prediction accuracy of OS. The calibration curve showed consistency of OS at 1, 3, and 5 years (Fig. 5B).

### Correlation between HCC risk score and immune-related gene expression

**Fig. 6:**
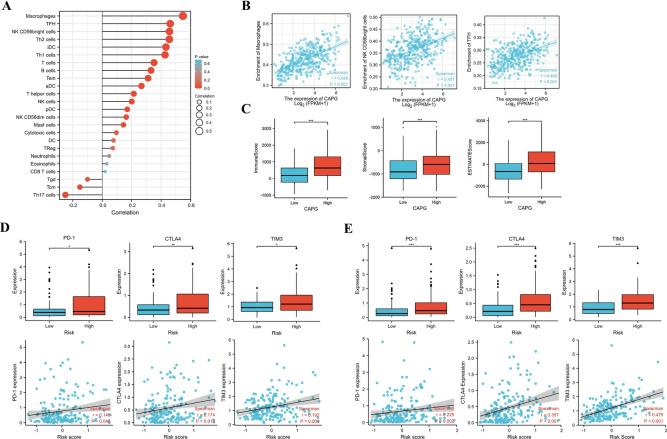
Correlations between the risk score and immune-related genes and the immune cell landscape in HCC **A** and **B** The lollipop diagram shows the difference between 24 infiltrated immune cells and the expression of CAPG in the tumor microenvironment. The scatter plots show the enrichment of the top three cells. **C** The box plots show the correlation between the expression of CAPG and immune, stromal and ESTIMATE scores. **D** and **E** Correlation between the risk score and the expression of PD-1, CTLA-4, and TIM3 in the training (left) and validation groups (right)

By analysing three key genes, we found that the expression of CAPG was highly correlated with various cellular immune microenvironment components (Fig. 6A). TFH cells, NK cells, CD56bright cells, and macrophages were the most correlated (Fig. 6B). There were significant differences in the immune score, stromal score, and ESTIMATE score between the high and low CAPG expression groups (Fig. 6C).

Additionally, both the training and validation groups assessed PD-1, CTLA-4, and TIM3 in patients with different risk scores. The expression levels of PD-1, CTLA-4, and TIM3 were higher in the high-risk group, and there was a positive correlation between the expression of these immune checkpoint genes and the risk score (Fig. 6D,E). Validation in LIRI-JP further confirmed our findings (Supplementary Fig. S2).

### CAPG is critical for HCC cancer cell proliferation

**Fig. 7:**
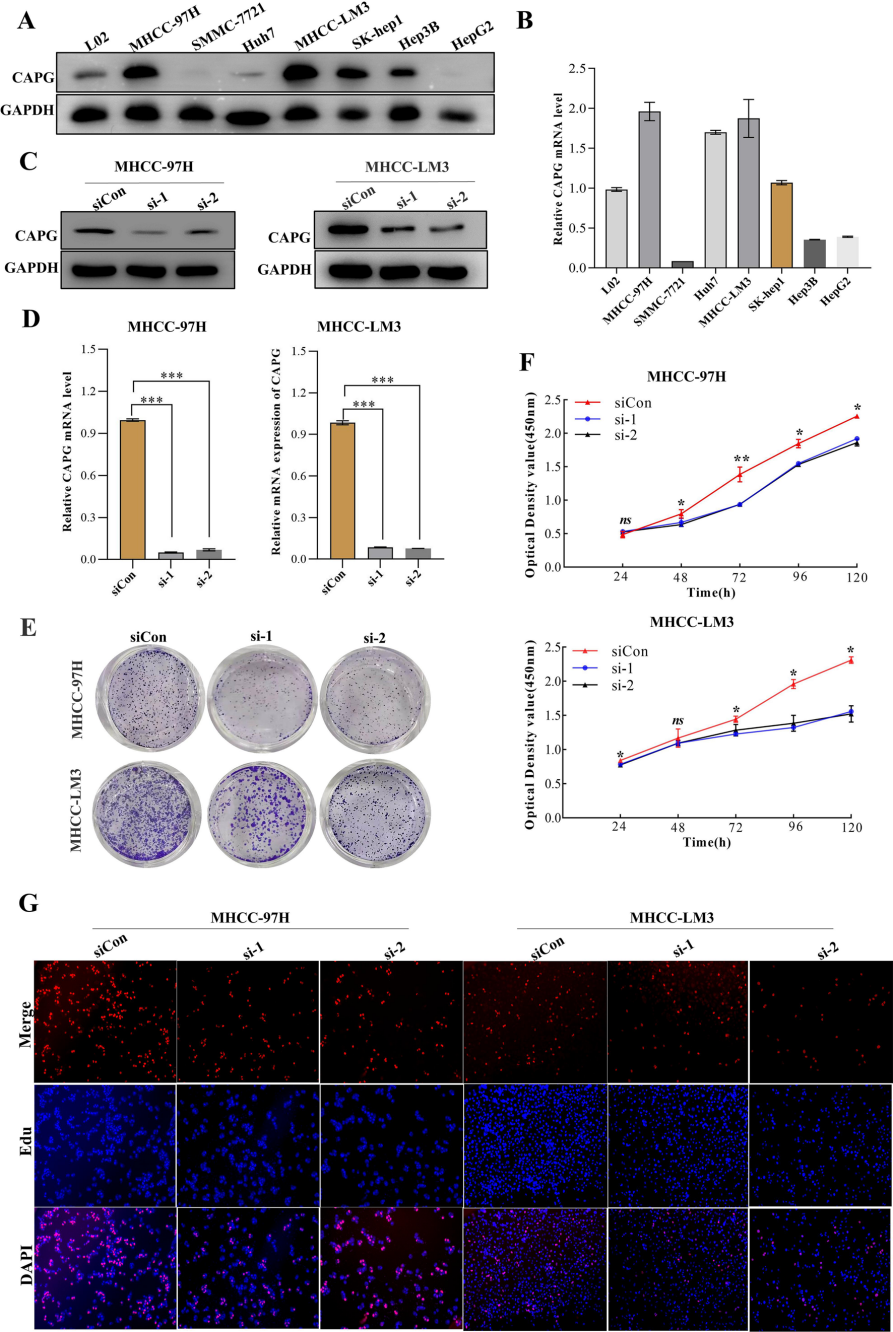
The effect of CAPG knockdown on the proliferation ability of HCC cell lines. **A** Protein levels of CAPG were determined in HCC cell lines by Western blotting. GAPDH was used as the internal reference. **B** RT‒PCR was carried out to evaluate the expression of CAPG in HCC cell lines. **C** Protein levels of CAPG were detected in the MHCC-97H and MHCC-LM3 cell lines after CAPG silencing (control-siRNA as a control; siCon). **D** RT‒PCR was carried out to evaluate the expression of CAPG in the MHCC-97H and MHCC-LM3 cell lines after CAPG silencing. **E** Proliferation ability was evaluated by colony formation assay in CAPG knockdown cell lines. **F** Cell viability was measured by CCK-8 assays in CAPG knockdown cell lines. **G** Cellular replication was assessed by EdU incorporation assays in CAPG knockdown cell lines

CAPG expression was investigated by quantitative RT‒PCR and Western blotting in a panel of established HCC cell lines. Among various cell lines, MHCC-97H and MHCC-LM3 cells showed the highest CAPG expression and were selected for further study (Fig. 7A, B).

To assess the effect of CAPG on HCC cell growth, we used two small interfering RNAs (siRNAs) to silence CAPG expression in MHCC-97H and MHCC-LM3 cell lines (Fig. 7C, D). CCK8, colony formation, and EdU incorporation assays indicated that the knockdown of CAPG markedly reduced cancer cell proliferation compared to that observed in the negative control (Fig. 7E–G). These results show that CAPG is critical for HCC cell proliferation.

### CAPG enhances cell proliferation by regulating ferroptosis via SLC7A11-mediated GSH synthesis

**Fig. 8:**
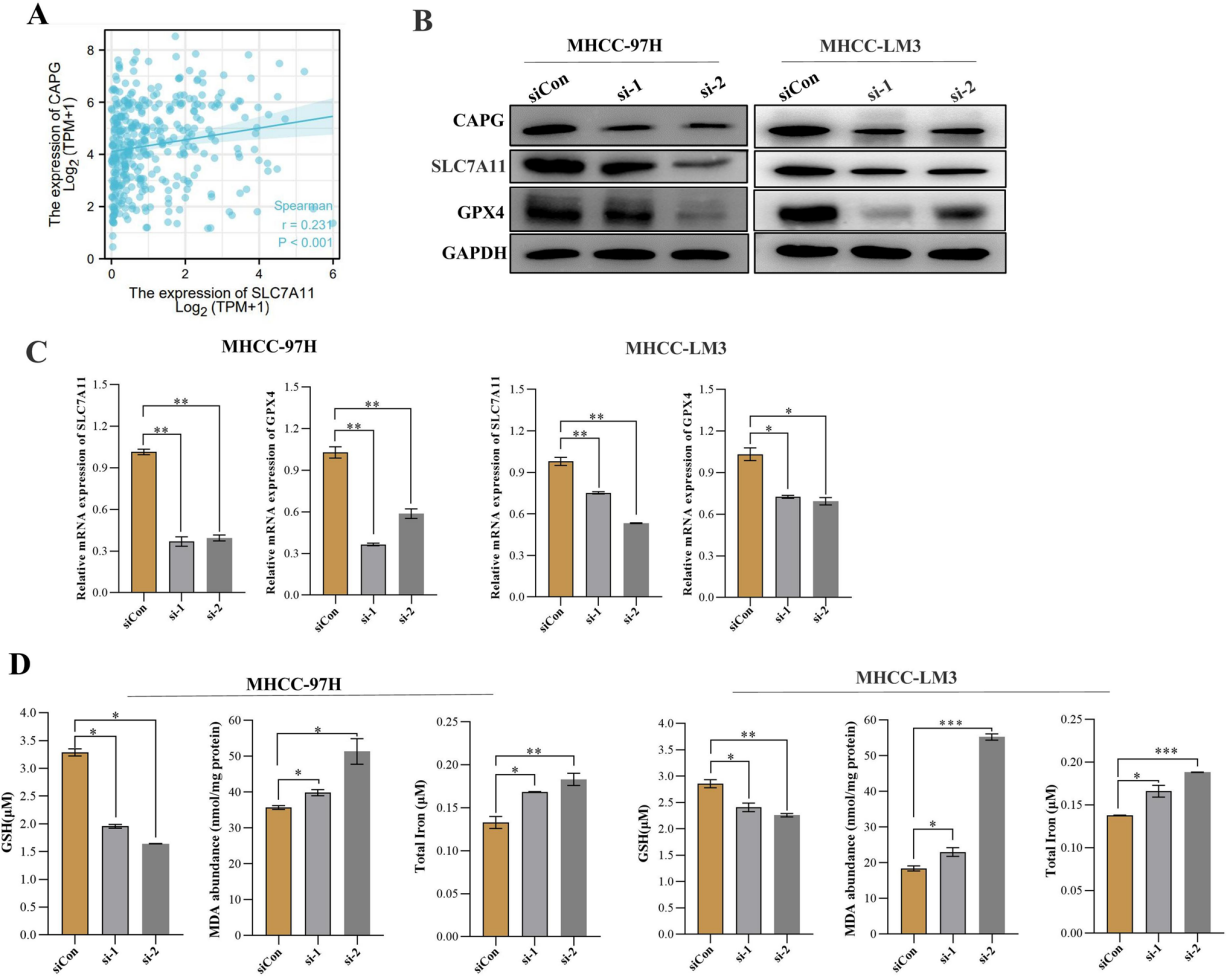
CAPG enhances cell proliferation by regulating ferroptosis via SLC7A11-mediated GSH synthesis. **A** Spearman correlation analysis between the expression of CAPG and SLC7A11 in HCC patients according to TCGA databases. **B** Protein levels of ferroptosis-related proteins were measured by Western blotting in CAPG knockdown cell lines. **C** The expression of ferroptosis-related proteins was detected by RT‒PCR in CAPG knockdown or overexpression cell lines. **D** GSH, MDA, and total iron were detected in the CAPG knockdown cell line

To study the role of CAPG in regulating ferroptosis in HCC, we examined the expression levels of key ferroptosis pathway components. First, bioinformatics analysis indicated that the expression of CAPG was positively correlated with the expression of SLC7A11 (Spearman r = 0.231* P* < 0.001; Fig. 8A). Western blotting and qRT‒PCR experiments indicated that CAPG knockdown markedly downregulated SLC7A11 levels in MHCC-97H and MHCC-LM3 cells (Fig. 8B, C). In addition, the level of GSH was decreased, while total iron and MDA were increased after CAPG knockdown (Fig. 8D). Collectively, these data show that CAPG enhances cell proliferation by regulating SLC7A11-mediated GSH synthesis and inhibiting ferroptosis in HCC.

## Discussion

In this study, we systematically explored the role of the ferroptosis-related gene signature in HCC. A prognostic model containing 3 ferroptosis-related genes was first constructed and validated in the TCGA-LIHC database. In addition, immune analysis, including various bioinformatics tools, revealed significant differences in the tumor microenvironment and immune cell infiltration between the low-risk and high-risk groups, particularly the immune checkpoints PD-1, CTLA-4, and TIM3. These findings strongly suggest the great potential roles of ferroptosis in HCC.

The high recurrence rate of HCC seriously affects the prognosis, but there is no effective prevention method [14]. With the development of systemic therapy, an increasing number of drugs have appeared after sorafenib, but the improvement in survival time is still unsatisfactory [15]. Immunotherapy based on immune checkpoints has recently been used for HCC; however, its efficiency is limited to less than 20% [16, 17]. Therefore, it is urgent to study the development, progression, recurrence, and metastasis mechanism of HCC, which will help to reveal the key regulatory pathways or networks in cancer and promote the development and improvement of complementary therapies. Ferroptosis, a newly identified nonapoptotic regulated cell death driven by excessive lipid peroxidation, has been implicated in cancer development and therapeutic responses [7, 10]. Dysregulation of ferroptosis-associated regulators, including different “drivers”, “suppressors”, and “markers”, is involved in all stages of tumor development [18]. This study used TCGA transcriptome data to explore the potential regulatory mechanism and prognostic value of ferroptosis-related genes in HCC. Forty-nine ferroptosis-related genes were differentially expressed in HCC tumors, and 3 ferroptosis-inhibiting genes (CAPG, SLC7A11, SQSTM1; upregulated in HCC) with poor prognosis were identified by univariate Cox and multivariate Cox regression analyses. The ferroptosis-based risk score can accurately judge the prognosis of patients. Notably, the risk score was also identified as an independent prognostic factor for HCC. Additionally, we developed a highly accurate prognostic map for predicting the 1-year, 3-year, and 5-year OS of HCC, which was higher than that of the C-index in a previous study [19].

Of note, there was a significant correlation between ferroptosis-associated genes and immune-related genes in HCC. Given the important supporting role of the tumor microenvironment in tumorigenesis and development, tumor malignant behavior is also regulated by other cells and factors in the microenvironment [1, 20]. The interaction among tumor cells, immune cells, and the immunosuppressive microenvironment regulates tumor progression in various stages [21]. Therefore, ferroptosis-mediated changes in tumor biological behavior may play a role through immune-related mechanisms [10]. In this study, we found that the expression of PD-1, CTLA-4, and TIM3 was significantly different between the two groups. When further constructing the risk score model based on three key genes related to ferroptosis, the PD-1, CTLA-4, and TIM3 immune checkpoint genes were highly expressed in the high-risk groups. These findings suggest the potential role of ferroptosis in regulating immune checkpoint expression in the tumor microenvironment and provide potential guidance for HCC patients to choose immunosuppressive therapy.

Some studies have explored the roles of SLC7A11, CAPG, and SQSTM1 in cancer [7, 22–25]. SLC7A11 (XCT) is one of the most important inhibitory pathway molecules in ferroptosis, and most inducers of ferroptosis, such as erastin and sorafenib, target SLC7A11 [7, 26]. It should be noted that SLC7A11 is significantly expressed in poorly differentiated HCC tissues [22], which is consistent with our findings, and the SLC7A11 inhibitor SASP enhances ROS-mediated apoptosis in CDDP-treated HCC cells. The gelsolin-like actin-capping protein CAPG is a member of the calcium-sensitive actin-binding protein family, which plays an important role in regulating cytoplasmic and nuclear structures [27]. Studies have reported that CAPG, as a tumor-promoting gene, may mediate the occurrence and development of various cancers [23, 28, 29]. The overexpression of CAPG in the cytoplasm of human HCC is associated with cell invasion, migration, and tumor prognosis in HCC [24]. However, the mechanisms underlying the functional roles of CAPG in HCC have rarely been reported to date. In our study, CAPG was significantly upregulated in HCC cells, and the knockdown of CAPG markedly reduced cancer cell proliferation, suggesting that CAPG is critical for HCC cell proliferation. We also found that CAPG knockdown markedly downregulated SLC7A11 levels. In addition, the level of GSH was decreased, while total iron and MDA were increased after CAPG knockdown. These results indicated that CAPG might enhance cell proliferation by regulating SLC7A11-mediated GSH synthesis and inhibiting ferroptosis in HCC. Further studies are still needed to explore the specific mechanisms of CAPG involvement in HCC.

There are several limitations in our study. First, the sample size in our study was quite small, and more datasets are needed to further confirm our results. Second, we did not conduct clinical validation in our patients to further evaluate the prognostic value of the model established in our study. Finally, we only conducted an in vitro experiment to explore the potential role of CAPG in HCC development. The specific molecular mechanisms by which CAPG regulates SLC7A11 are currently unknown and will be the focus of our future work.

In summary, the risk prediction model for individual prognosis of HCC was developed according to the risk score of ferroptosis-related genes and vital clinical information, which may be clinically used in early diagnosis, diagnosis assistance, and decision-making adjuvant therapies of HCC. Specifically, CAPG may drive HCC progression by regulating the SLC7A11-GSH ferroptosis signaling pathway.

## Supplementary Information

Additional file 1—The raw data of TCGA-LIHC.

Additional file 2—The raw data of three GEO datasets.

Additional file 3—The raw data of LIRI-JP from ICGC.

Additional file 4—Table of 49 DEGs with log2FC and p-value.

Additional file 5—Fig. S1 External Validation of Figure 4 in ICGC.

Additional file 6—Fig. S2 External Validation of Figure 6 in ICGC.

Additional file 7—Fig. S3 Uncropped images of all blots. Uncropped images of Figure 7A, Figure 7C, Figure 8B, were shown.

Additional file 8

## Data Availability

The datasets generated and analysed during the current study are available in TCGA (https://portal.gdc.cancer.gov/) and GEO (https://www.ncbi.nlm.nih.gov/geo/). Further inquiries can be directed to the corresponding authors.

## Abbreviations

HCC: Hepatocellular carcinoma
CAPG: Capping actin protein, gelsolin‒like
SLC7A11: Solute carrier family 7 member 11
SQSTM1: Sequestosome 1
log_2_FC: Log_2_ fold change
GEO: Gene expression omnibus
TCGA: The cancer genome atlas
AUC: Areas under the curves
ROC: Receiver operating characteristic
OS: Overall survival
ROS: Reactive oxygen species
FPKM: Fragments per kilobase per Million
RNA-seq: RNA sequencing
DEGs: Differentially expressed genes
PPI: Protein‒Protein interaction
GO: Gene ontology
KEGG: Kyoto encyclopedia of genes and genomes
KM: Kaplan‒Meier
ssGSEA: Single sample gene set enrichment analysis
TIP: Tumor immunophenotype
qRT‒PCR: Quantitative real-time PCR
mRNA: Messenger RNA

## Code availability

All statistical analyses were conducted by R software version 4.2.1 (www.xiantao.love). Concurrently, Metascape, a gene annotation and analysis resource, and GraphPad Prism (version 8.0.1) also participated in the analysis.
