# Supplementary figures and images for: Comprehensive analysis of ferroptosis-related genes for clinical and biological significance in hepatocellular carcinoma
Source: Discov Oncol. 2023 May 17;14:69. doi: 10.1007/s12672-023-00677-4 (PMC10192498; doi:10.1007/s12672-023-00677-4)

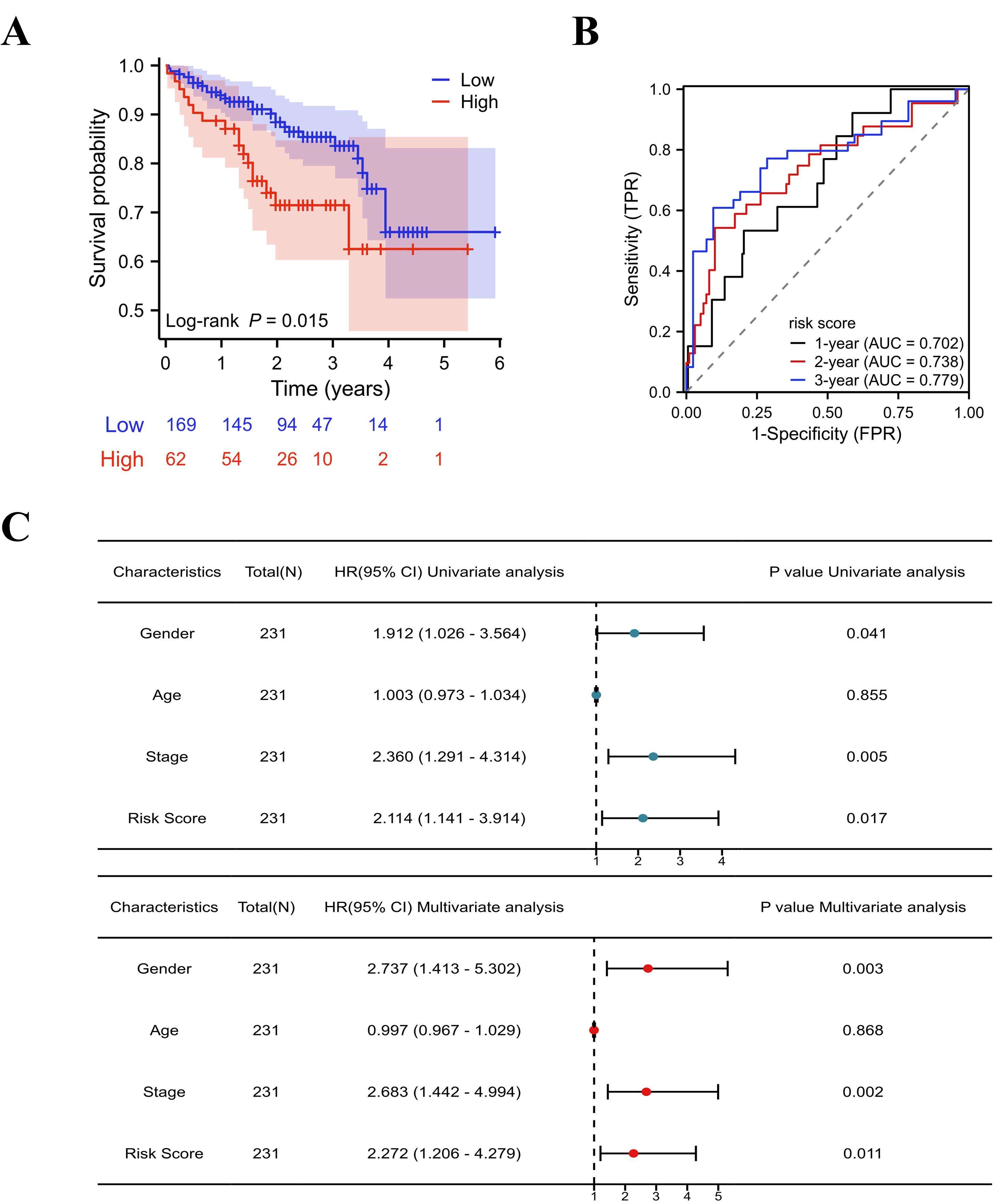

Supplement: Supplementary file 5 — Additional file 5—Fig. S1 External Validation of Figure 4 in ICGC. [file 12672_2023_677_MOESM5_ESM.jpg]

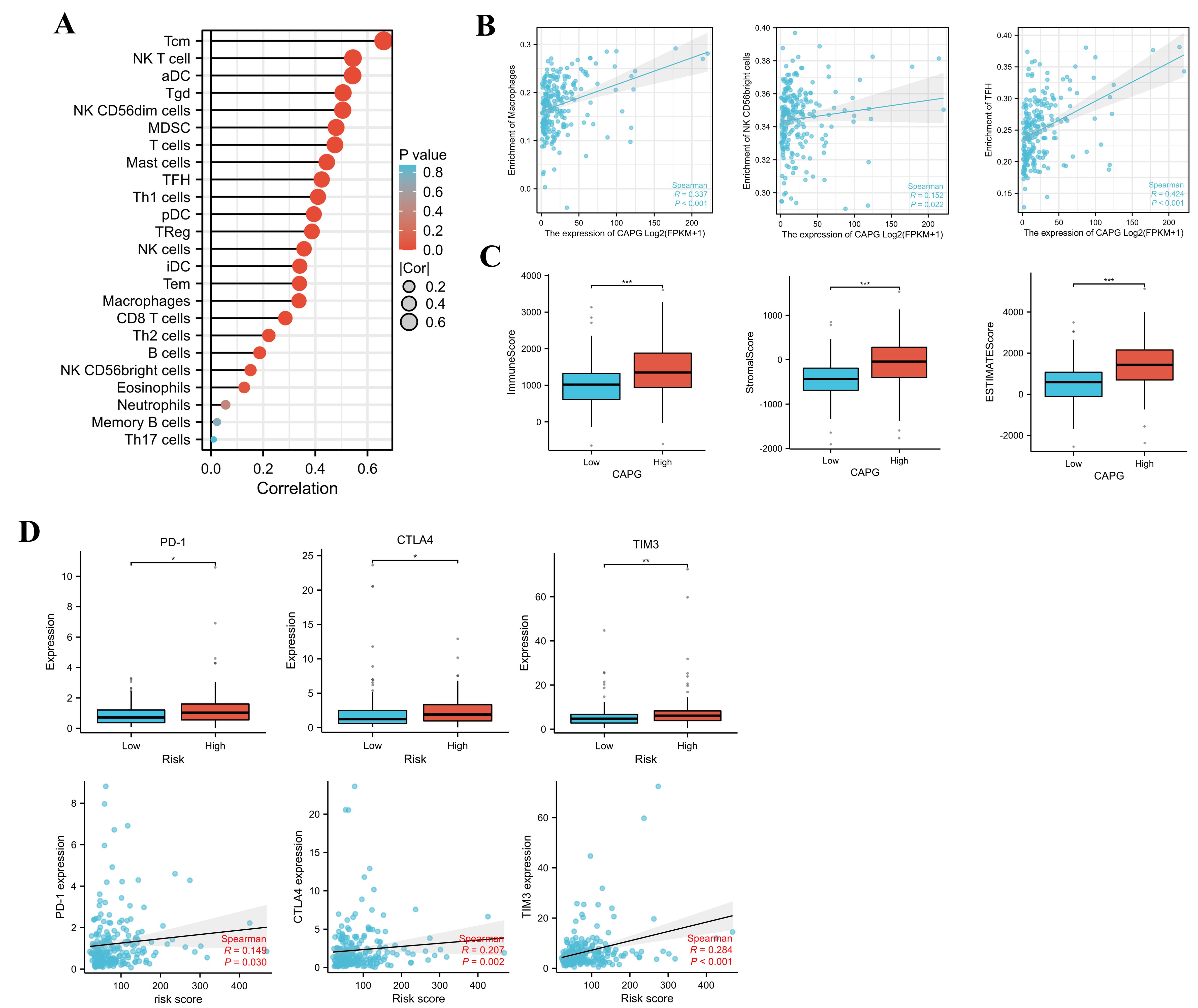

Supplement: Supplementary file 6 — Additional file 6—Fig. S2 External Validation of Figure 6 in ICGC. [file 12672_2023_677_MOESM6_ESM.jpg]
