# Supplementary material for: Comprehensive analysis of ferroptosis-related genes for clinical and biological significance in hepatocellular carcinoma
Source: Discov Oncol. 2023 May 17;14:69. doi: 10.1007/s12672-023-00677-4 (PMC10192498; doi:10.1007/s12672-023-00677-4)
Supplement: Supplementary file 7 — Additional file 7—Fig. S3 Uncropped images of all blots. Uncropped images of Figure 7A, Figure 7C, Figure 8B, were shown. [file 12672_2023_677_MOESM7_ESM.docx]

**Comprehensive Analysis of Ferroptosis-Related Genes for Clinical and Biological Significance in Hepatocellular Carcinoma**

**Qixian Wu ^1, 2, 4^, Zhenlin Tan ^1, 4^, Yu Xiong ^3^, Chengxin Gu ^1^, Jingdon Zhou ^2^, Hui Yang ^1, *^, Jiyuan Zhou ^1, *^**

1. Department of Gastroenterology, the Second Affiliated Hospital of Guangzhou Medical University, Guangzhou, China
2. Guangzhou Medical University, Guangzhou, China
3. Intervention and Cell Therapy Center, Peking University Shenzhen Hospital, Shenzhen, China
4. These authors contributed equally to this work.

**^*^Corresponding author:**

Jiyuan Zhou, M.D., Ph. D

E-mail: jyzhou03@bjmu.edu.cn

Hui Yang, M.D., Ph. D

E-mail: yanghui@gzhmu.edu.cn**Additional file Figure 3**

**A**

**
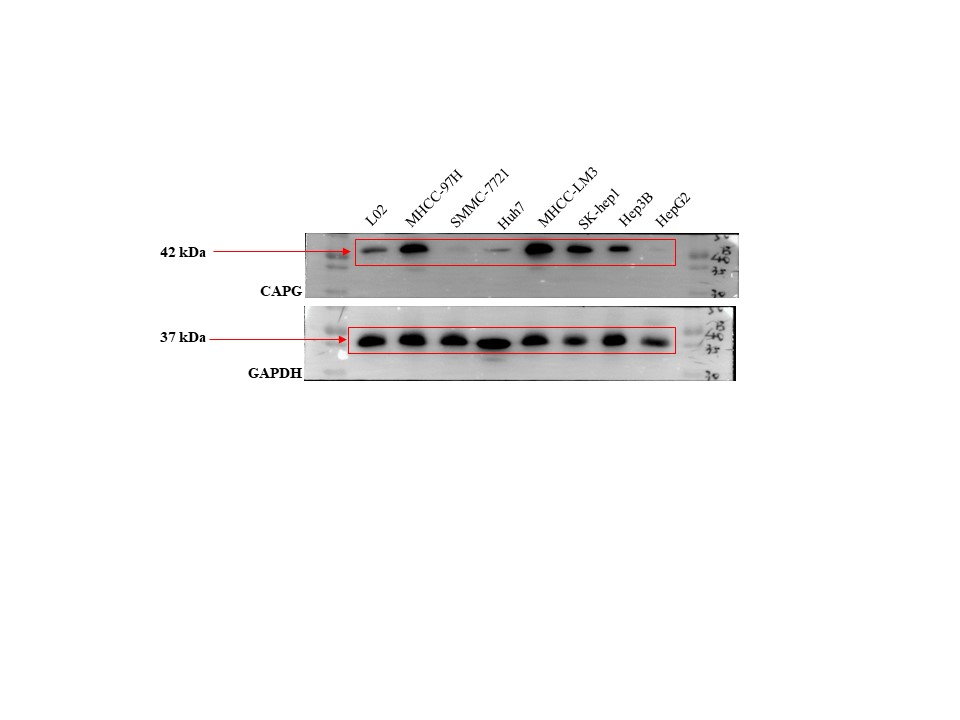
**

**B**

**
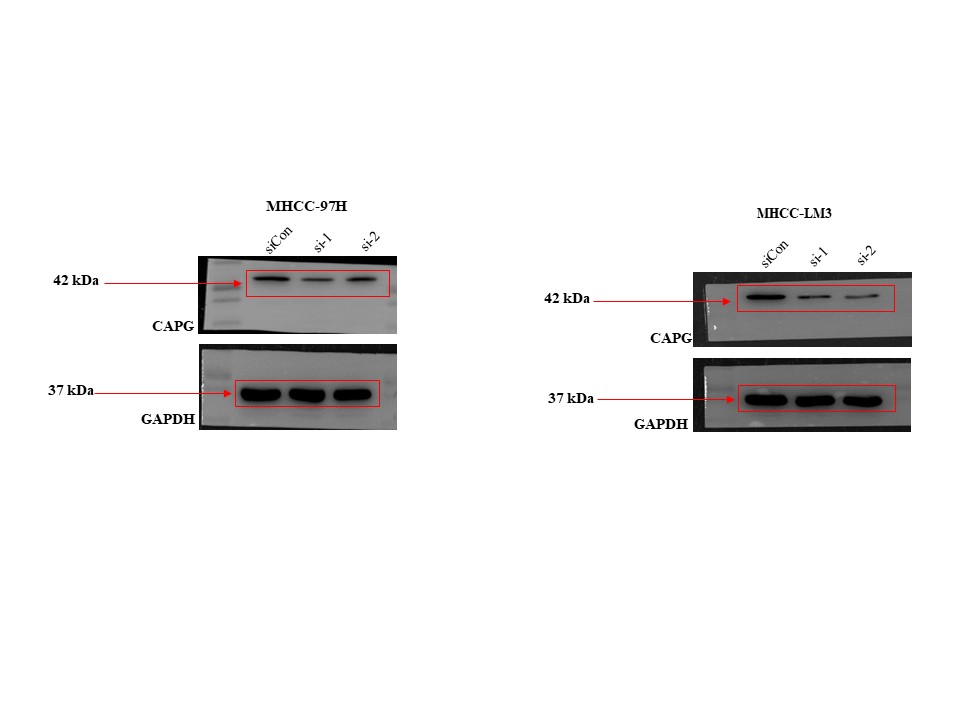
**

**C**

**
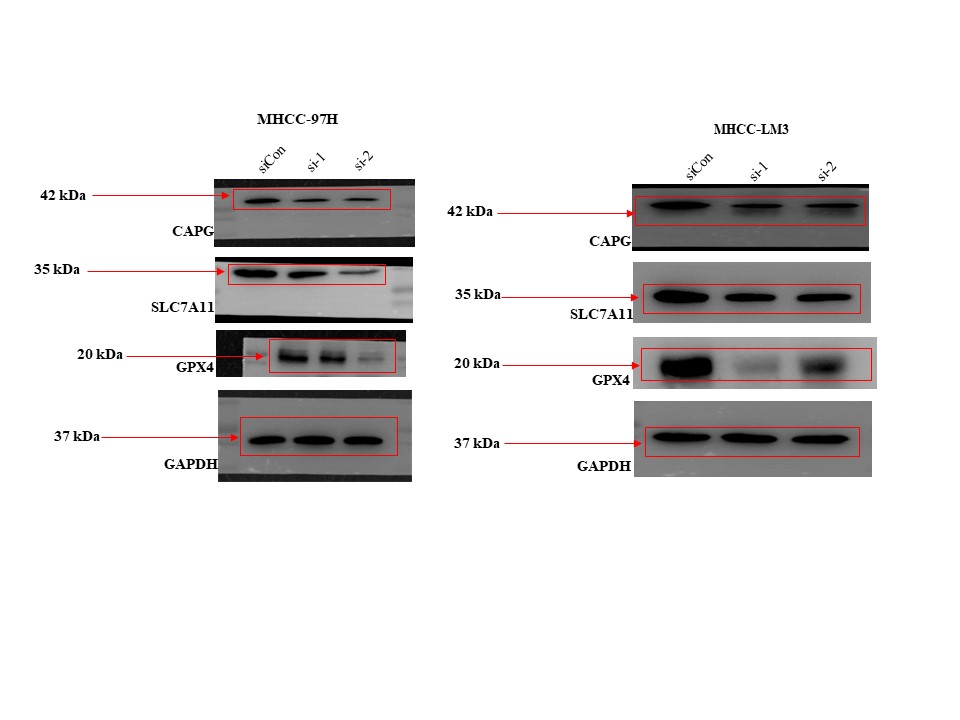
**
