# Supplementary material for: Comprehensive analysis of ferroptosis-related genes for clinical and biological significance in hepatocellular carcinoma
Source: Discov Oncol. 2023 May 17;14:69. doi: 10.1007/s12672-023-00677-4 (PMC10192498; doi:10.1007/s12672-023-00677-4)
Supplement: Supplementary file 8 — Additional file 8 [file 12672_2023_677_MOESM8_ESM.docx]

# Supplementary materials

**Table S1. The information of datasets from the GEO database**

| **Accession number** | **Species** | **Platform** | **Samples** | **Experiment type** | **PMID** |
| --- | --- | --- | --- | --- | --- |
| GSE25097 | Homo sapiens | GPL10687 | 557 | expression profiling by array | 200025097 |
| GSE36376 | Homo sapiens | GPL3106 | 433 | expression profiling by array | 200036376 |
| GSE45267 | Homo sapiens | GPL570 | 87 | expression profiling by array | 200045267 |

**Table S2. Primers for RT-PCR**

| **Name** | **Forward primer (5'-3')** | **Reverse primer (5'-3')** |
| --- | --- | --- |
| CAPG | CGAACACTCAGGTGGAGATT | TCCAGTCCTTGAAAAATTGC |
| SLC7A11 | TTACCAGCTTTTGTACGAGTCT | GGTCCTCTTCTCCGCCTCCTTC |
| GPX4 | CGATAAGGATCAACCCCATACA | GTGAGCTTGCAAAAGGTTAAGA |
| GAPDH | ATGGTTAACCTGGACAAGTACC | GACGAGCTGAGTGTAGTTTACT |
